# Immunotherapeutic Efficacy of Retargeted oHSVs Designed for Propagation in an Ad Hoc Cell Line

**DOI:** 10.3390/cancers13020266

**Published:** 2021-01-12

**Authors:** Andrea Vannini, Valerio Leoni, Mara Sanapo, Tatiana Gianni, Giorgia Giordani, Valentina Gatta, Catia Barboni, Anna Zaghini, Gabriella Campadelli-Fiume

**Affiliations:** 1 Department of Experimental, Diagnostic and Specialty Medicine, University of Bologna, 40126 Bologna, Italy; valerio.leoni2@unibo.it (V.L.); mara.sanapo4@unibo.it (M.S.); tatiana.gianni3@unibo.it (T.G.); valentina.gatta6@unibo.it (V.G.); 2 Department of Pharmacy and Biotechnology, University of Bologna, 40126 Bologna, Italy; giorgia.giordani3@unibo.it; 3 Department of Veterinary Medical Sciences, University of Bologna, 40126 Bologna, Italy; catia.barboni@unibo.it (C.B.); anna.zaghini@unibo.it (A.Z.)

**Keywords:** oncolytic virus, herpes simplex virus, retargeted virus, tropism retargeting, tumor, immunotherapy, checkpoint inhibitor, vaccination, antigen-agnostic vaccination, HER2

## Abstract

**Simple Summary:**

The onco-immunotherapeutic viruses, among which stand the onco-immunotherapeutic herpes simplex viruses, have gained renewed interest due to their ability to unlock the potential of checkpoint inhibitors in preclinical and clinical settings. In prior decades, safety concerns led to the generation of overall safe, partially or highly attenuated oncolytic viruses. Current focus is on more efficacious onco-immunotherapeutic viruses with limited ability to cause off-tumor and off-target infections and the capability to subvert the tumor microenvironment immunosuppression—hence to potentiate checkpoint inhibitors. These viruses might serve as potential partners of T-cell therapies.

**Abstract:**

Our laboratory has pursued the generation of cancer-specific oncolytic herpes simplex viruses (oHSVs) which ensure high efficacy while maintaining a high safety profile. Their blueprint included retargeting to a Tumor-Associated Antigen, e.g., HER2, coupled to detargeting from natural receptors to avoid off-target and off-tumor infections and preservation of the full complement of unmodified viral genes. These oHSVs are “fully virulent in their target cancer cells”. The 3rd generation retargeted oHSVs carry two distinct retargeting moieties, which enable infection of a producer cell line and of the target cancer cells, respectively. They can be propagated in an ad hoc Vero cell derivative at about tenfold higher yields than 1st generation recombinants, and more effectively replicate in human cancer cell lines. The R-335 and R-337 prototypes were armed with murine IL-12. Intratumorally-administered R-337 conferred almost complete protection from LLC-1-HER2 primary tumors, unleashed the tumor microenvironment immunosuppression, synergized with the checkpoint blockade and conferred long-term vaccination against distant challenge tumors. In summary, the problem intrinsic to the propagation of retargeted oHSVs—which strictly require cells positive for targeted receptors—was solved in 3rd generation viruses. They are effective as immunotherapeutic agents against primary tumors and as antigen-agnostic vaccines.

## 1. Cancer-Selective Oncolytic Herpes Simplex Viruses and Synergism with Check Point Blockade

Herpes simplex viruses (HSVs) were among the first viruses taken into consideration as candidate oncolytic viruses (OVs) [1,2,3,4] and still rank high in the list of OVs in clinical trials [5]. The early approaches to generate OVs, including oHSVs, were rather conservative. Safety was a major concern, probably because scientists wanted to avoid the problems which characterized the initial approaches to gene therapy, and because of the frailty of patients. Most of the OVs that entered the clinical trials were attenuated, or over-attenuated. In reality, safety proved not to be a major clinical issue. oHSVs, and OVs in general, are well tolerated in clinical applications with very limited description of serious adverse effects [6,7]. However, the efficacy in humans did not keep up to the expectations raised by animal experimentation. For most OVs, and particularly for oHSVs, attenuation has been the prerequisite for cancer selectivity, and hence for safety. Cancer cells exhibit varying defects in innate responses and weakly contrast viral replication. Attenuated viruses exploit such defects to target cancers cells, which sustain the replication of both wt and attenuated viruses, and to spare non-cancer cells, in which the replication of attenuated viruses is contrasted, but not fully abolished, by the antiviral innate response. One such example is the attenuation conferred by the deletion of the γ34.5 virulence gene [2,3,4]. Since attenuation also weakens virus replication in cancer cells, additional modifications were introduced in the Δγ34.5 oHSVs to rescue replication and virulence. The α47 open reading frame was deleted to augment antigen presentation. The deletion modified the expression profile of the late US11 gene. Immunomodulatory genes were engineered in the viral genome. This is essentially the genotype of the approved OncovexGM-CSF, also known as T-VEC or Imlygic^®^ [2,8].

The interest in OVs, including oHSVs, was boosted by check point inhibitors (CPIs) [9]. These molecules disable the breaks imposed by some tumors on the anti-cancer immune machinery and unleash the T-cell response against tumors. In humans, CPIs are limited by the fact that they target a restricted range of tumors—typically those with high mutational load, high tumor-specific T-cell infiltration and low T-cell activity due to the checkpoint brakes—and are effective only against a fraction of patients. The rationale for combining oncolytic viruses, like OncovexGM-CSF, with checkpoint inhibitors is compelling [10,11,12,13]. OVs inflame tumors, overcome the tumor microenvironment (TME) immunosuppression, and unlock the potential of checkpoint inhibitors across many cancer types. The underlying mechanism rests on the direct oncolysis induced by virus replication and consequent increase in both total and cancer-specific antigenic load in the TME, on the ability to recruit the immune cells to TME and to induce the expression of pro-inflammatory molecules. In turn, the latter activate immune effector cells and cause tumor inflammation. Given the heterogeneity in cancer genotypes and phenotypes, it is foreseen that even more complex combinations of immunomodulatory agents may be required to obtain consistent and durable therapeutic responses against a broad spectrum of cancers [14,15]. oHSVs are well equipped to do this job, since their large genome has space for extra genes. GM-CSF is a potent pro-inflammatory cytokine, most frequently employed as a payload in oHSVs [5]; it targets mainly the myeloid lineage, activates the dendritic cells, and enhances anti-cancer effects; it is present in OncovexGM-CSF. IL-12 (interleukin-12) [16] is another highly effective pro-inflammatory cytokine transgenically expressed by OVs, since it orchestrates the innate and the adaptive immune response against cancer and pathogens [17]. It is present in the oHSV named M032, currently in clinical trial [18,19]. Additional payloads recently engineered in oHSVs include the ligands to co-stimulatory immune receptors, CPI or combinations thereof [20,21].

## 2. Strategies towards Cancer-Specific and Efficacious oHSVs

In recent years efforts were made to generate cancer-specific rather than cancer-selective oHSVs. Such viruses gain safety from specificity, contain the entire set of viral genes so that they counteract the antiviral innate response, replicate robustly in the tumor cells, and are effective in releasing the immune suppression typical of cancers and in reactivating tumor recognition by the immune system.

### 2.1. Tropism Retargeting

The notion of tropism retargeting was pioneered by Glorioso and co-workers, and by Roizman and Zhou, and has been reviewed [22,23,24,25]. It entails the genetic engineering of a ligand to a selected cancer receptor into one of the viral glycoproteins that mediate HSV entry into the cells, most frequently gD [24,26,27]. Crucial to this development has been the elucidation of the molecular events that govern HSV entry. It requires four essential glycoproteins, gD, gH, gL and gB, which are activated in a cascade fashion, and two major alternative receptors HVEM (herpesvirus entry mediator) and nectin1 which interact with gD and activate it; they serve as major tropism determinants. Thereafter, the receptor-activated gD and additional integrin receptors activate the heterodimer gH/gL, and finally gB. The latter executes the fusion between the virion envelope and the cell membranes—plasma membranes or endocytic membranes [28,29,30,31,32]. The presence of cell surface receptors is a requirement for HSV entry [33].

**Figure 1:**
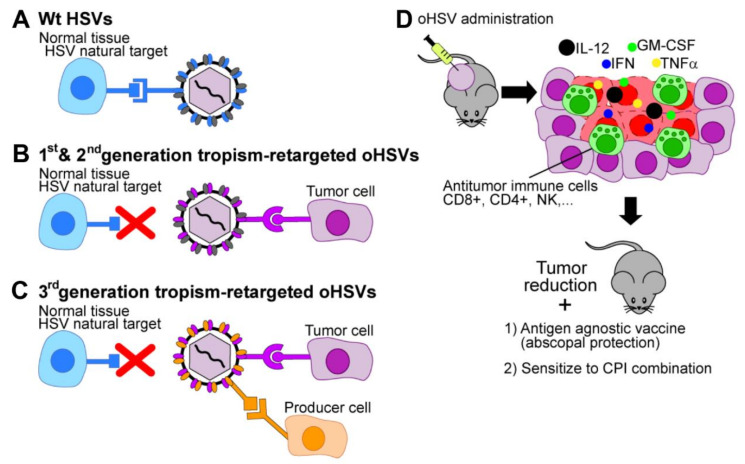
Schematic view of HSV tropism retargeting and immunotherapy induced by armed oHSVs. (**A**–**D**). (**A**) wt-HSV infects normal tissues, i.e., its natural target cells through natural major receptors HVEM and nectin1 (blue). (**B**) 1st and 2nd generation tropism retargeted oHSVs infect cancer cells expressing the target Tumor Associated Antigen (TAA, violet) and fails to infect its natural targets. The scFv to TAA which mediated the virus retargeting to cancer cells is engineered in the following virion glycoproteins: gD (1st generation), either gB or gH (2nd generation). (**C**) 3rd generation tropism retargeted oHSV, can infect both the cancer cells that express the target TAA (violet) and the producer cells that express an artificial receptor (orange), and fails to infect its natural targets. (**D**) The IL-12-armed retargeted oHSV specifically infects tumors cells, causes immunogenic cell death of cancer cells and elicits immuno-therapeutic response, that result in inhibition of tumor growth, sensitization to checkpoint blockade and antigen-agnostic vaccination.

The strategy developed in our laboratory to generate cancer-specific oHSVs and increase their efficacy entails (a) the retargeting of the virus tropism to Tumor-Associated Antigens (TAAs), i.e., to molecules that are selectively present at cancer cell surfaces; (b) the detargeting of the virus tropism from the natural receptors HVEM and nectin, to avoid off-target and off-tumor infections. When combined, retargeting and detargeting provide specificity and safety; (c) preservation of the full spectrum of viral genes, to enable a robust anti-tumor response [26,27]. Since the retargeted oHSVs do not carry any genetic modification in virulence or other genes, they are “fully virulent viruses in their target cancer cells” (Figure 1). Clearly, the extent of cancer-specificity reflects the specificity of the target to which the oHSVs are addressed. Some TAAs—such as EGFRVIII (epidermal growth factor receptor Variant III), IL-13 Receptor 2-α and HER2 (human epidermal growth factor receptor 2) are more specific than others. A further improvement was introduced by Glorioso and coworkers through point mutations in gD that impair the binging of neutralizing antibodies, and thus make the oHSV stealth to anti-HSV antibodies present in the human population [34].

Bench and preclinical studies foresee the following advantages for the tropism retargeted oHSVs. In contrast to the Δγ34.5 oHSVs that can replicate solely in cancer cells that carry defects in certain pathways of the innate response and may potentially replicate in non-cancerous cells defective in innate responses, the retargeted oHSVs infect cancer cells irrespective of the status of their innate response and are designed to prevent off-target infections. The extent of infections in tissues with low level expression of the targeted receptors remains to be verified in humans. Moreover, once the retargeted oHSVs infect the tumor cells, they are essentially wt-viruses, they blunt the cell innate response and promote high viral replication [35,36,37,38]. Lastly, in the tumor bed, the retargeted oHSVs exclusively infect the cancer cells, whereas the Δγ34.5 and the attenuated viruses additionally infect immune cells, with unclear effects on the global immune response.

### 2.2. Transcriptional and Post-Transcriptional Retargeting Strategies

Additional strategies to attain cancer specificity and preserve viral virulence include transcriptional retargeting—i.e., placing a critical viral gene under the control of a cancer-specific promoter, post-transcriptional retargeting and combinations thereof [39]. Examples include the control of the key γ34.5 virulence gene by a hybrid nestin enhancer-HSP68 minimal promoter which ensures expression of the HSV genome specifically in the nestin-positive glioblastoma cells [40] and the insertion of miRNA target sequences specific for selected tissues (e.g., brain, heart, or liver) to avoid off-tumor expression of key herpesviral proteins like ICP4 (infected cell protein 4), ICP27, UL8, and γ34.5 [20]. Such approaches have been elegantly reviewed recently [23] and are beyond the scope of current review.

## 3. Properties of the Tropism-Retargeted oHSVs Generated in Our Laboratory

### 3.1. Three Generations of Tropism Retargeted oHSVs

For heuristic reasons we divide the retargeted oHSVs generated in our laboratory into three groups (Table 1). In all, the retargeting was achieved by insertion of a single chain antibody (scFv) to the receptor of choice, while detargeting was achieved by deletion of appropriate portions in gD [27,41] [WO2009144755] (Table 1)

The 1st generation recombinants carry the scFv in gD, in place of either aa 6-38 or aa 61–218. Such deletions eliminate the portions in gD responsible for interactions with HVEM and nectin1, and confer full detargeting. In different recombinants, the scFvs were addressed alternatively to HER2 (human epithelial growth factor receptor 2), EGFR (epithelial growth factor receptor), EGFRVIII (EGFR variant III) or PSMA (prostate specific membrane antigen) [27,42] and WO2009144755.

The 2nd generation recombinants carried the scFv to HER2 or to EGFR in either gH or gB. This recombinant group explored the possibility that glycoproteins essential for HSV entry, other than gD, serve as vector for the scFv. They carry the Δ6–38 in gD [43,44] and WO201612849.

The 3rd generation recombinants simultaneously carry two retargeting moieties. The rationale is detailed below (see, paragraph 3.3). One moiety is the anti-HER2 scFv inserted in gD for cancer cell retargeting. The other moiety is the GCN4 peptide engineered alternatively in gD, gH or gB for retargeting to an ad hoc producer cell line. For detargeting purposes, the 3rd generation recombinants contained one of the following deletions in gD: aa 6–38, two single amino acids—ΔD30 and ΔY38, or deletions in the nectin binding site encompassing aa 214–223 [44,45,46,47] [WO2017211941, WO2017211944, WO2017211945].

### 3.2. The Retargeted oHSVs are a Platform

TAAs constitutes a family of molecules, with varying degrees of cancer specificity. Very often, the encoding genes are genetically amplified in cancer cells, such that the TAAs are overexpressed in cancer cells, and poorly or not expressed in non-cancerous cells. Many are located on the cell surface. Since each member of the family is expressed across several cancer types [48], a single retargeted oHSV can potentially be employed against a number of different cancers. In most of our studies we selected HER2, expressed and amplified in a number of cancers, including breast, ovary, stomach, lung and pancreas cancers and glioblastoma, and is a relevant target in cancer immunotherapy. Thus, a HER2-retargeted oHSV can potentially be employed against a variety of indications. Glorioso laboratory, as well as our additionally generated oHSVs retargeted to EGFR, EpCAM, EGFRVIII specific for glioblastoma puntiforme, and PSMA, present in prostate cancers [23,26,49,50,51]. The EGFRVIII recombinant was further improved by insertion of a matrix metalloproteinase which enhanced intratumoral vector distribution and efficacy in a glioblastoma model [52]. Essentially, the retargeted oHSVs are a platform and can potentially be addressed to different TAAs. It is envisioned that the intensive molecular profiling programs carried out worldwide may lead to the discovery of novel TAAs, even more specific than the ones currently known.

### 3.3. Cultivation of Tropism-Retargeted oHSVs in Non-Cancerous Producer Cells

**Figure 2:**
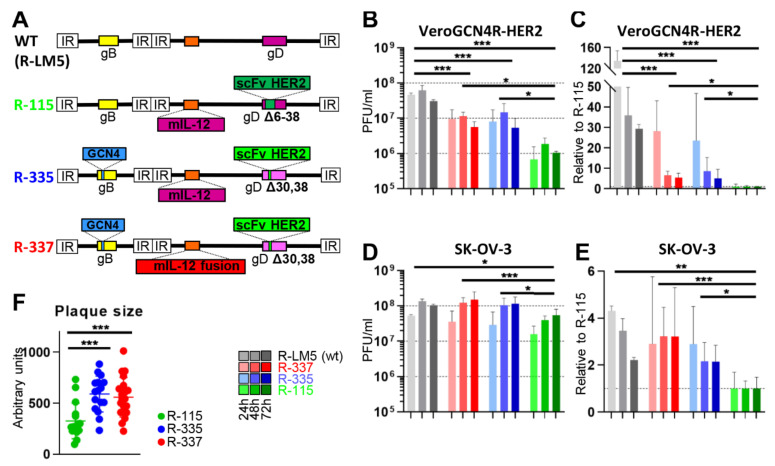
Growth kinetics of 1st and 3rd generation HER2-retargeted oHSVs in cancerous and non-cancerous producer cells. (**A**) Schematic representation of genomes of wt HSV named R-LM5 (carrying GFP), R-115 (1st generation), R-335 and R-337 (3rd generation) retargeted oHSVs. Indicated are the genetic loci of gB, gD, the insertion site of mIL-12 in the US1 and US2 intergenic locus. 1st and 3rd generation recombinant viruses carry the insertion of scFv anti-HER2 for the retargeting to HER2-positive cells, and the deletion of indicated portions of gD for the detargeting from HSV-1 natural receptors HVEM and nectin1. The 3rd generation R-335 and R-337 viruses carry the GCN4 peptide in gB between aa 81 and 82, and were engineered as detailed in [17,54]. (**B**–**E**) Yields of the wt HSV named R-LM5 (carrying GFP), R-115, R-335 and R-337 in VERO-GCN4R-HER2 (**B**,**C**) and in SK-OV-3 (**D**,**E**) cells at 24, 48 and 72 h after infection. Replicate cultures of each cell line were infected with the indicated viruses at 0.1 PFU/cell as titrated in SK-OV-3 cell line. Progeny virus was titrated in SK-OV-3 cells. In panels C and E, yields are expressed relative to that of R-115. The data represent the average of at least five independent experiments ± SD. (**F**) Plaque size of the indicated viruses five days after infection. For each virus-infected culture 20 plaque pictures were taken, expressed as arbitrary units and plotted ± SD. (**B**–**F**) Statistical significance was calculated by One Way ANOVA test and expressed as * = *p*-value < 0.05; ** = *p*-value < 0.01; *** = *p*-value < 0.001.

A critical feature of the retargeted oHSVs generated in our laboratory is that they are strictly dependent on the targeted cancer receptor for infection, including infection of the producer cells. Often, the target receptor is an oncogene, i.e., it contributes to the oncogenic potential of the cancer cells. While the retargeted oHSVs can be readily cultivated in human cancer cells lines positive for the targeted receptor, approval of clinical grade virus production in cancer cells by competent authorities might likely imply specific motivations. A goal in the design of the 3rd generation retargeted oHSVs was to generate a non-cancerous producer cell line for the in vitro growth of the retargeted oHSVs. To this end, as mentioned above, we designed recombinants that simultaneously carry two retargeting moieties (Figure 1 and Figure 2A). The scFv retargets HSV to the cancer receptor (HER2, in our case). The second one consists of a small high affinity ligand (GCN4 peptide) engineered in one of the entry glycoproteins—gD, gH or gB (Figure 2A) [45,46,47]. The producer cell line is a Vero cell derivative, named Vero-GCN4R-HER2, which expresses an artificial receptor for the GCN4 peptide, along with human HER2.

In subsequent sections of this review, we shall focus on the two most advanced recombinants from the 3rd generation group, R-335 and R-337. They carry (i) the insertion of the GCN4 peptide in gB between residues 81 and 82; (ii) the deletion of only two amino acids in gD—D30 and Y38—for HVEM and nectin1 detargeting; (iii) the insertion in gD of scFv to HER2 in place of Y38. R-335 and R-337 carry mIL-12 in the US1/US2 intergenic region, a site that enables a high expression level. While R-335 carried the natural form of mIL-12, made of p40 and p35 subunits, R-337 carried the fusion form, in which the two subunits are held together by a linker to form a single peptide. In cultured cells, the fusion form was produced at 50 to 100-fold higher amounts than the dimeric form and possibly was more stable. R-335 and R-337 genotypes are depicted in Figure 2A. The properties of OVs, and particularly oHSVs expressing IL-12 are reviewed in [53].

Preliminarily, we quantified the ability of the two recombinants to grow in Vero-GCN4R-HER2 and in the human HER2-positive cancer cell line SK-OV-3. The yields of R-335 and R-337 in Vero-GCN4R-HER2 are shown in Figure 2B, which also shows the yields of R-LM5 (an essentially wt HSV carrying EGFP [27]) and of the 1st generation recombinant R-115. R-335 and R-337 infect these cells through both the GCN4R and HER2 receptors, whereas R-115 infects only through HER2. Figure 2C shows the fold-increase of the yields relative to that of R-115. Two features emerged. R-335 and R-337 replicated to seven to eight-fold higher yields than R-115. As expected, R-335 and R-337 replicated to fivefold lower yields than R-LM5; this is a common feature for recombinant viruses and accounts for different receptor usage, in that R-LM5 infection occurs through the simian orthologs of the natural receptors nectin1 and HVEM, which ensure the best possible interaction for HSV entry into the cells.

In our experience the highest yields for both wt-HSV and HER2-reatargeted oHSVs are obtained in SK-OV-3. Figure 2D,E show that the wt R-LM5 grew somewhat better in SK-OV-3 than in Vero-GCN4R-HER2 cells, as expected. The growth of R-335 and R-337 could not be differentiated from that of R-LM5 and was about three-fold higher than that of R-115. In addition, R-335 and R-337 plaques in SK-OV-3 cells were doubled in size relative to those from R-115, in agreement with the virus yields results (Figure 2F). The higher replication of R-335 and R-337 relative to that of R-115 was surprising in that SK-OV-3 cells lack the receptor for GCN4 peptide. We interpret these results to indicate that the smaller deletion in gD improved the glycoprotein performance, and that the GCN-4 insertion in gB somehow activated gB or a combination of these effects.

### 3.4. Safety Profile of Retargeted oHSVs

**Figure 3:**
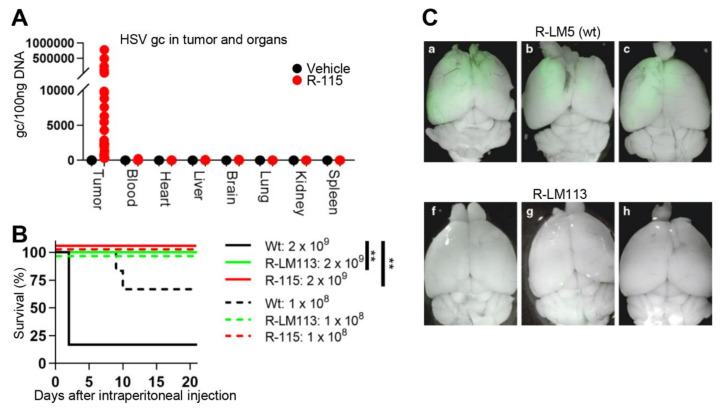
The retargeted oHSVs are safe in mice upon intraperitoneal, intratumoral or intracranial routes of administration. (**A**) R-115 biodistribution to the indicated organs following four intratumoral injections (1 × 10^8^ PFU/dose or vehicle), started at d 10 after tumor implantation. Organs and tumors were explanted at d 26, and, after homogenization, the total DNA was extracted. R-115 genome copy numbers were determined by qPCR in comparison with a standard curve obtained with purified HSV DNA, and expressed as gc/100 ng of DNA or gc/100 µl blood. (**B**) Kaplan Meier survival curves of the C57BL/6 mice intraperitoneally injected with 1 × 10^8^ or 2 × 10^9^ PFU of R-LM5 (wt HSV), R-LM113 and R-115 (1st generation) oHSVs. (**C**) Merged fluorescence and bright-field images of adult nonobese diabetic/severe combined immunodeficient (NOD/SCID) mouse brains after injection with R-LM5 (1 × 10^5^ PFU) or R-LM113 (3 × 10^5^ PFU) viruses. Viral spread is visualized by enhanced green fluorescent protein fluorescence. (**A**) Statistical significance was calculated using the Log-rank (Mantel-Cox) test. Panels (**A**–**C**), reproduced with permission. ** = *p*-value < 0.01.

Safety of retargeted oHSVs rests on their specificity for cancer cells and on genetic stability and is documented by the following lines of evidence. In vitro, both 1st and 3rd generation retargeted oHSVs infected almost exclusively the cancer cells positive for the targeted receptor and failed to infect or infected very poorly receptor-negative cancer cells and non-cancerous cells, unless they transgenically expressed the targeted receptor [27,41]. In no cases did the infection of few cells in a culture of receptor-negative cells result in a virus that could be serially passaged. The viruses exhibit genetic stability in that they have been passaged in cultures for several months (3rd generation) or years (1st generation), without any change in retargeting/detargeting properties. In vivo, upon intratumoral (i.t.) administration, the 1st generation R-115 was detectable only in the tumors, and not in serum or other organs (Figure 3A) [55]. When administered intraperitoneally (i.p.), the 1st generation oHSVs did not cause any pathological signs, including brain infections, even at the highest amounts (2 × 10^9^ PFU). Under the same conditions, the wt-HSV killed all mice (Figure 3B) [41,56]. In vivo, upon intracranial administration, the 1st generation R-LM113 virus did not infect the brains, whereas the wt-HSV readily did (Figure 3C) [57]. Altogether, the results support the notion that (i) that in vitro infection of human cells only occurs at high level HER2 expression, and (ii) the HER2-retargeted oHSVs do not cause detectable off-target infections in mice. A detailed analysis on bio-distribution to human tissues, especially in tissues with low level HER2 expression, remains to be performed.

## 4. In Vivo Efficacy of Retargeted oHSVs in Immunocompetent Mouse Models

### 4.1. Efficacy against LLC-1-HER2 Primary Tumors

**Figure 4:**
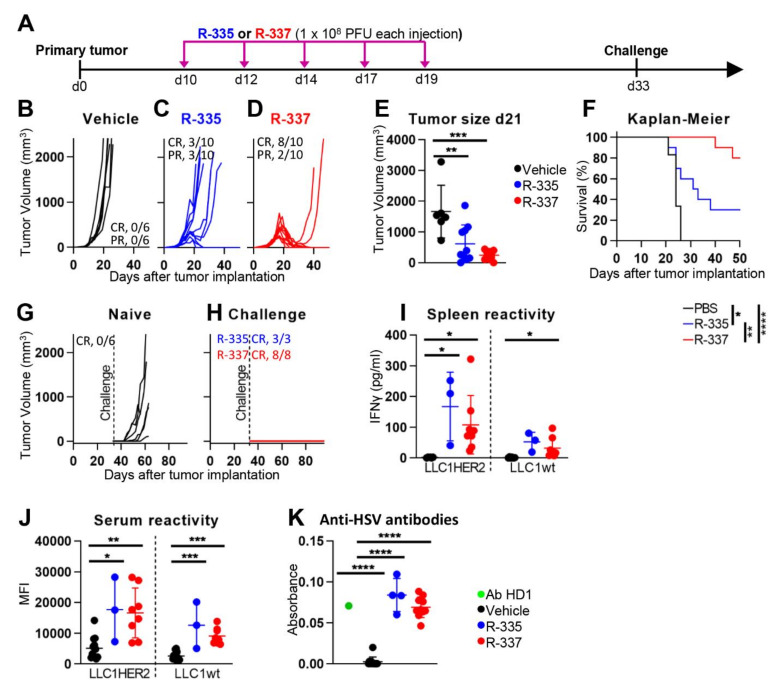
Efficacy of R-335 and R-337 monotherapy on the growth of LLC-1-HER2 tumors. (**A**) Schedule of treatments. The six-to-eight weeks old HER2-transgenic/tolerant (HER2-TG) C57BL/6 mice were subcutaneously implanted in the left flank with 5 × 10^5^ LLC-1-HER2 cells in 100 μL of PBS [55]. 10 d later, when the tumor volumes averaged 70–100 mm^3^, mice received 5 intratumoral injections of R-335, R-337 (1 × 10^8^ PFU per injection, diluted in 50 μL PBS) or vehicle (50 μL PBS), at 2–3 day intervals. At d 33, the mice which survived the primary tumor received a contralateral challenge LLC-1-HER2 tumor of 5 × 10^5^ cell per mouse. Tumor volume was calculated using the formula: largest diameter x (smallest diameter) 2 × 0.5. Mice were sacrificed when tumor volumes exceed 1000–2000 mm^3^, ulceration occurred, or animals exhibited distress or pain. (**B**–**D**) Kinetics of tumor growth in mice treated with vehicle (**B**), R-335 (**C**) or R-337 (**D**). The numbers reported in each panel indicate the numbers of mice which were completely cured from tumors (complete response, CR), or which showed a delay/reduction in tumor growth (partial response, PR). The mice were scored PR when the tumor volume was <50% smaller than the mean size of the tumors in the vehicle group. (**E**) Volumes of the primary tumors at d 21 after implantation. Black (vehicle), blue (R-335) and red (R-337) circles. (**F**) Kaplan-Meier survival curves of the three groups of mice. (**G**,**H**) Kinetics of growth of contralateral challenge tumor in naïve mice (**G**), and in the R-335 or R-337 (**H**) arms. (**I**) Immune response in splenocytes harvested at sacrifice. To isolate splenocytes, spleens were smashed through a 70 µm cell strainer in PBS, red blood cells were lysed with ACK buffer, and samples were resuspended in medium (RPMI 1640 containing 10% heat inactivated FBS, 1% penicillin/streptomycin). Splenocytes (1 × 10^6^ cell/well) were incubated with 1 × 10^5^ LLC-1-HER2 or LLC-1 cells in 0.5 mL medium, and cocultured for 48 h. The amount of secreted IFNγ (quantified by ELISA) was a measure of the splenic anti-LLC-1 and anti- LLC-1-HER2 immune response [55]. (**J**,**K**) Antibody reactivity in sera harvested at sacrifice to LLC-1-HER2 or LLC-1 cells (**J**), and to HSV-1-infected cells (**K**), as determined by cell enzyme-linked immunosorbent assay (CELISA). Wt-LLC-1 and LLC-1-HER2 single cell preparations were reacted with mouse serum, diluted 1:150 in flow cytometry buffer (PBS + 2% FBS), in ice for 1 h, washed with flow cytometry buffer and incubated with anti-mouse PE (1:400). Data were acquired on BD C6 Accuri. For CELISA assay, RS cells were infected with HSV-1 at 3 PFU/cell for 24 h, then they were fixed with paraformaldehyde, reacted with mouse serum diluted 1:60, or with the anti-gD monoclonal antibody HD1 (green) diluted 1:400 (positive control), followed by anti-mouse peroxidase. Peroxidase substrate o-phenylenediamine dihydrochloride was added and plates were read at 490 nm as detailed [55]. (F) Statistical significance was calculated by the Log-rank (Mantel-Cox) test. (**E**,**I**–**K**) Statistical significance was calculated by means of the One Way ANOVA test and expressed as * = *p*-value < 0.05; ** = *p*-value < 0.01; *** = *p*-value < 0.001; **** = *p*-value < 0.0001. Color code: mice which received Vehicle, R-335 or R-337 are indicated in black, blue or red, respectively.

Early studies from our laboratory indicated that retargeted oHSVs are highly effective in nude mice, a property which only accounted for direct oncolytic effects [41,56]. The key question arose as to how effective the retargeted oHSVs are in immunocompetent mice, in particular in eliciting the innate response to the virus, the innate and the adaptive long-term immunity to the tumor. To address this question here we provide the first description of the efficacy of R-335 and R-337 and review previously described efficacy data on 1st generation recombinants R-LM113, R-115 and R-123. A list of the most significant preclinical studies carried out in our laboratory is reported in Table 2. As discussed above, the HER2-retargeted oHSVs are strictly dependent on (human) HER2 to carry out infection, a feature that required an ad hoc immunocompetent murine model. Preliminarily, we screened a number of murine tumor cell lines and found that LLC-1 (Lewis lung carcinoma-1) cells enabled the highest HSV replication [55]. The ad hoc model consists of LLC-1 cells made transgenic for HER2 (LLC-1-HER2) and of the syngeneic C57Bl/6 mice transgenic and tolerant (TG) to HER2. Mouse tolerance to HER2 was critical to prevent that the immune response to the tumor was mainly driven by the allogeneic HER2 [55]. With respect to antitumor activity, the 1st generation R-115 protected 60% of mice, of which 16% exhibited a complete response (CR) and 44% a partial response (PR) [55]. The experimental design in anti-tumor efficacy experiments is illustrated in Figure 4, panel A. Briefly, mice were implanted with subcutaneous tumors; the recombinants were administered intratumorally (i.t.) to well-developed tumors. The mice that survived the primary tumor received a second challenge tumor, which was untreated. The R-335 and R-337 recombinants were administered i.t. to well-developed tumors as five injections of 1 × 10^8^ PFU each, every other day (Figure 4A). The antitumor activity of R-335 was similar to that of R-115, while that of R-337 was higher. In particular, R-335 protected 60% of mice, 30% of which exhibited a complete response (CR) (Figure 4B–E). R-337 protected 100% of the mice, 80% of which exhibited CR (Figure 4B–E). The Kaplan Meier survival curve shows highly statically significant differences between each virus and the control, and between the two viruses (Figure 4F). The superior efficacy R-337 relative to R-335 should be interpreted in light of the fact the only genotypic difference between the two viruses resides in mIL-12, which is a heterodimer in R-335 and a fusion form in R-337. The results clearly indicate that a significant contribution to the control of primary tumor growth is immune mediated.

### 4.2. Retargeted oHSVs Promote Antigen Agnostic Vaccination Effect against Distant Tumors

A notable property of R-115 is the long-term abscopal efficacy [55]. Mice which survived the primary tumor were fully protected from a distal tumor implanted at later times. Essentially, R-115 vaccinated mice against a subsequent challenge tumor. Even the 3rd generation R-335 and R-337 proved to be particularly effective. Of the mice described in Figure 4C,D, those which survived the primary tumor received a challenge LLC-1-HER2 tumor 33 days later. All these mice were fully protected (Figure 4G,H). Inasmuch as the mice did not receive any treatment after the implantation of the distant challenge tumor, any protection seen against such tumors was immune-mediated.

The mice protected from distant tumors exhibited a T-cell immune response documented as splenocyte reactivity to tumor cells (Figure 4I), in agreement with similar finding with R-115 [55]. In particular, the splenocytes from both R-335- and R-337-treated mice, harvested at sacrifice, reacted strongly to LLC-1-HER2 cells, and weakly to LLC-1 cells (Figure 4I). The antibody response reflected at large the T-cell response, in that the sera from R-335- and R-337-treated mice carried antibodies to LLC-1-HER2 and, to a lesser extent, to LLC-1 cells (Figure 4J). The extent of protection against distant wt-LLC-1 tumors will be evaluated in detail in future studies. Current results argue that the intratumoral treatment of LLC-1-HER2 tumors with R-335 or R-337 can elicit a protective response also to wt-LLC-1 cell neoantigens. The mice sera showed seroconversion also to HSV-1 (Figure 4K), as expected.

### 4.3. Retargeted oHSVs Subvert TME Immunosuppression

**Figure 5:**
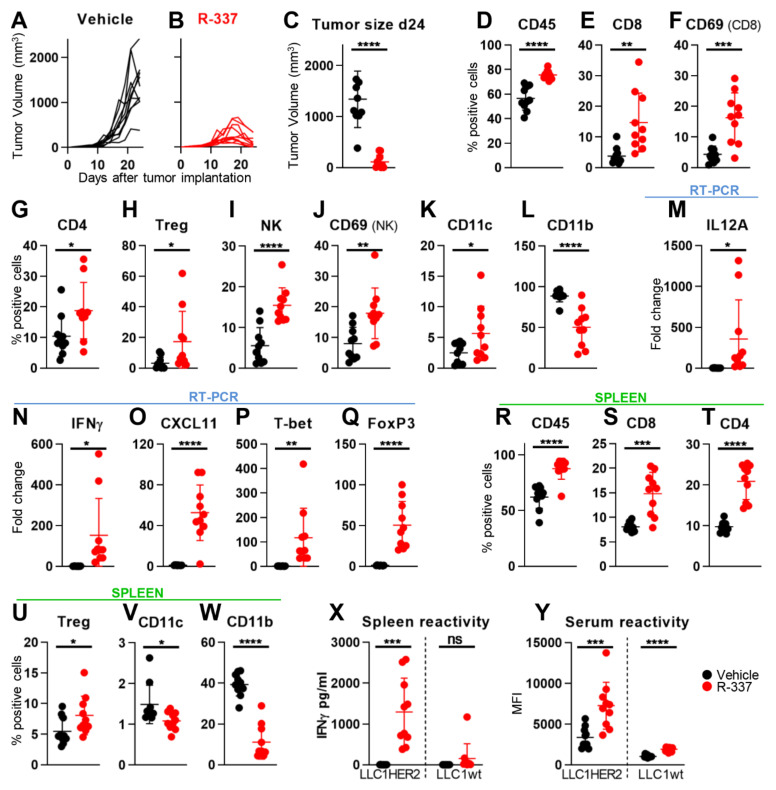
Immune heating of TME and spleen modifications induced by intratumoral R-337 monotherapy. (**A**,**B**) Kinetics of tumor growth in HER2-TG C57BL/6 mice treated with vehicle (**A**) or R-337 (**B**), according to the schedule reported in Figure 4A. (**C**) Tumor volumes at d 24. Black (vehicle) and red (R-337) circles. (**D**–**L**) Immune cell populations in tumors. Single cell suspensions were prepared from freshly isolated LLC1-HER2 tumors at sacrifice. Tumors were minced in small pieces, digested with collagenase, passed through 70 μm cell strainer and rinsed with FACS buffer. For each sample, 2 × 10^6^ cells were blocked with α-CD16/32 Ab (clone 93), and then reacted with the antibodies CD4-FITC (clone GK1.5), CD8a-PE (clone 53-6.7), CD45-Percp-Cy7 (clone 30-F11), CD335-APC (clone 29A1.4), FoxP3-PE (clone 150d/e4), CD11b-FITC (clone M1/70), CD11c-PE (clone N418) and CD69-PercP (clone H1-2F3). Data were acquired on BD C6 Accuri. CD4 (CD4+ cells), CD8 (CD8+ cells), NK (CD335+ cells) and myeloid cells (CD11b+ cells) were gated on CD45+ subpopulation. Activated (CD69+) CD8 and NK cells were gated on CD8+ and CD335+ subpopulations, respectively. DC cells (CD11c+CD11b+) were gated on CD11b+ population. Tregs (FoxP3+CD4+) were gated on CD4+ population. (**M**–**Q**). Expression profile of cytokines, immune related transcription factor and immune markers. Tumor homogenates (a few mgs) were employed for total RNA purification and 1.2 µg of RNA was employed for the cDNA synthesis. Diluted cDNAs (1:4) were assayed by real-time PCR with Taqman probes. The levels of expression were determined using the ΔΔCt method, normalized on the Rpl13a housekeeping gene and on the mean of the vehicle-treated group. (**R**–**W**) Immune cell populations in spleens. Sample preparation and staining as described for tumors. (**X**) Immune response in splenocytes to LLC-1-HER2 and LLC-1 cells was quantified as IFNγ secretion in the culture medium. For the details, see Figure 4. (**Y**) Serum antibody reactivity to LLC-1-HER2 and LLC-1 cells. For the details, see Figure 4C–Y Statistical significance was calculated by the t-test and expressed as * = *p*-value < 0.05; ** = *p*-value < 0.01; *** = *p*-value < 0.001; **** = *p*-value < 0.0001, ns = not significant. Color code, mice treated with vehicle or R-337 are indicated in black or red, respectively.

The purpose of this series of experiments was to provide evidence that the long-term distant protection was mediated by an immune response, documented as dramatic changes to the immunosuppressive TME. In these experiments, mice were treated i.t. with the R-337, and sacrificed a few days after the end of treatment, at a time when tumors were decreasing in size (Figure 5A–C). Analyses were carried out on tumor infiltrating lymphocytes and cytokines, on the reactivity of splenocytes and of serum antibodies to tumors cells, with the aim to detect local and systemic modifications. In R-115-treatred mice, the major modifications consisted in the tumor infiltration by CD4+, CD8+ and activated CD8+, NK (natural killer) and activated NK, Tregs (T-regulatory), along with the reduction in intratumoral CD11b+ leucocytes [55]. The immune landscape of LLC-1-HER2 TME is that of an immunologically desert tumor, characterized by low infiltration from anti-tumor immune subpopulations and low levels of immune activation markers, co-stimulatory molecules and pro-inflammatory cytokines [61]. In essence, the host immune system is unable to recognize and react against LLC-1 tumors. Figure 5D–K documents the modifications detected in R-337-treated mice. Worth noting are the increase in tumor infiltrating leucocytes, specifically CD4+, CD8+ and activated CD8+, DCs, and NK and activated NK cells. The CD11b-positive population, which includes the immunosuppressive myeloid derived suppressor cells, was decreased (Figure 5L). FoxP3+ cells, which include the T-regulatory cells, were also increased (Figure 5H), in agreement with previous reports [55]. Transcriptional analysis of the tumor specimens revealed an increase in IFNγ, IL-12 (most of which likely expressed from the viral genome), CXCL11 chemokine and t-bet transcription factor (Figure 5M–Q), hallmarks of inflamed TME and polarization to activated Th1 cells. Analysis of the systemic effect was carried out on spleen samples. The modifications were essentially similar to those detected in the tumor samples (Figure 5R–W), except that the increase in NK cells was non statistically significant. The splenocyte reactivity and the antibody response to LLC-1-HER2 cells were essentially similar to those detected in mice sacrificed at about 100 days after primary tumor implantation (Figure 5X,Y). Altogether, i.t.- administered R-337 elicited a strong systemic and intratumoral immune response, and the inflammation of the LLC-1-HER2 TME.

### 4.4. The “Immune Heating” of the Tumor Predisposes to Combination Therapy

**Figure 6:**
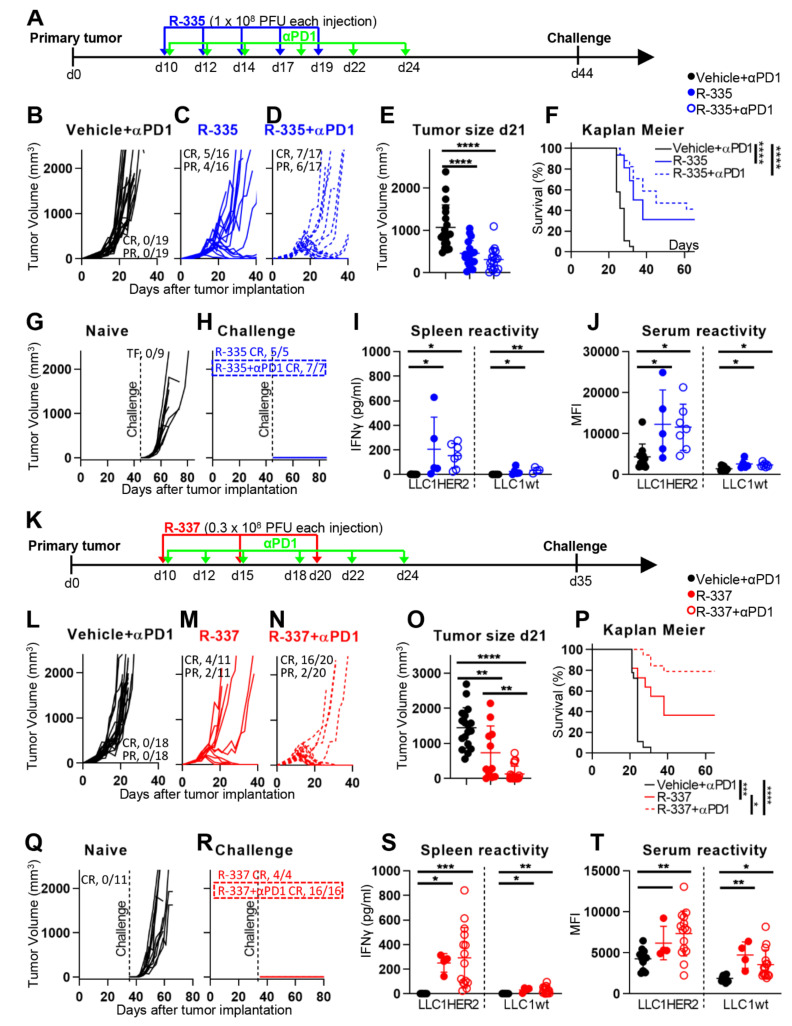
Efficacy of R-335 of R-337 in combination with anti-PD1 antibodies on the growth of LLC-1-HER2 tumors. (**A**) Schedule of the treatments. The HER2-TG C57BL/6 mice were implanted with LLC-1-HER2 cells. At d 10 after implantation, when tumors reached the average volume of 70–100 mm^3^, mice received 5 i.t. injections of R-335, or R-335 plus i.p. injections of anti-PD-1, at 2–3 days intervals. The administration schedule of oHSV and anti-PD-1 treatments was according to [62,63,64,65]. At d 44, the mice which survived the primary tumor received a contralateral challenge LLC-1-HER2 tumor. For the details, see Figure 4B–D Kinetics of tumor growth in mice treated with vehicle (**B**), R-335 alone (**C**), or R-335 plus anti-PD-1 combination therapy (**D**). Figures in panels indicate the number of mice exhibiting complete response (CR) or partial response (PR). (**E**) Volumes of the primary tumors at d 21 after implantation. Black (vehicle), blue (R-335) and open blue (combination) circles. (**F**) Kaplan-Meier survival curves of the three groups of mice. (**G**–**H**) Growth kinetics of contralateral challenge tumors in naïve mice (**G**), and in R-335 or combination arms (**H**). (**I**) Immune response in splenocytes harvested at sacrifice. Splenocytes were incubated with LLC-1-HER2 or LLC-1 cells. Activation was quantified as IFNγ secretion in the culture medium. (**J**) Serum antibody reactivity to LLC-1-HER2 or LLC-1 cells. (**K**) Schedule of the treatment with R-337 with or without combination with anti-PD-1 antibodies. The HER2-TG C57BL/6 mice were implanted with LLC-1-HER2 cells. At 10 d after tumor implantation, mice received 3 i.t injections of R-337 at 5 days interval, and, where indicated, i.p. injections of anti-PD-1 antibodies, as detailed in the drawing. At d 35, the mice which survived the primary tumor received a contralateral challenge LLC-1-HER2 tumor. (**L**–**T**) Kinetics of tumor growth (**L**–**N**), tumor size at d 21 (**O**), Kaplan Meier survival curves (**P**), growth curves of challenge tumors (**Q**–**R**), immune response in splenocytes (**S**), antibody reactivity to LLC-1-HER2 or LLC-1 cells (**T**). (**F**,**P**) Statistical significance was calculated by the Log-rank (Mantel-Cox) test. (E, I, J, O, S, T) Statistical significance was calculated by means of the ANOVA test and expressed as * = *p*-value < 0.05; ** = *p*-value < 0.01; *** = *p*-value < 0.001; **** = *p*-value < 0.0001. Color codes: mice treated with vehicle, R-335, R-337 are indicated in black, blue or red, respectively. Full circles and continuous lines, monotherapies. Open circles and dotted lines, combination therapies.

The distant long-term protection, along with the dramatic changes to TME induced by R-337, suggested that the recombinant could render immunologically cold and CPI-resistant tumors immunologically hot and possibly CPI-sensitive. LLC-1-HER2 tumors recapitulate tumors that are completely insensitive to anti-PD-1 (compare Figure 6B with Figure 4B), in agreement with the low immunogenicity of these tumors [61]. The experiment documented in Figure 6 was designed to ascertain whether R-335 and R-337 synergize with anti-PD-1. Mice were treated as in Figure 4, and additionally received anti-PD-1, administered i.p. (see Figure 6, panel A). It can be seen that, when combined with anti-PD-1 in a simultaneous regimen [62,63,64,65], R-335 displayed a tendency to increase efficacy (Figure 6B–E). Thus, in mice treated with R-335 alone, CR and PR occurred in 31 and 25% of mice, respectively, in agreement with data shown in Figure 4. In mice which received the combination therapy, CR and PR occurred in 41 and 35% of mice, i.e., 76% mice were protected, completely or partially. The Kaplan Meier survival curve is reported in Figure 6F. The mice which survived the primary tumor were fully protected from a challenge distant tumor (Figure 6G,H). The long-term protection was most likely based on the systemic immune response, documented as splenocyte and antibody reactivities to LLC-1-HER2 and wt-LLC-1 cells (Figure 6I,J).

To evaluate the efficacy of R-337 in combination with anti-PD-1, we decreased the overall amount of virus from five injections of 1 × 10^8^ PFU each to three injections of 0.3 × 10^8^ PFU each (in total, 0.9 × 10^8^ vs 5 × 10^8^) (Figure 6K). At this lower dosage, R-337 monotherapy induced CR in 36% mice and PR in 18%, with an overall response rate of about 55%. In the combination arm, 80% of mice exhibited CR, and 10% exhibited PR (Figure 6L–N). The difference between monotherapy and combination therapy was statistically significant with respect to tumor size (Figure 6O) and Kaplan Meier survival curve (Figure 6P). The surviving mice were fully protected from a distant challenge made of LLC-1-HER2 cells (Figure 6Q,R). At sacrifice, 80 days after primary tumor implantation, the mice treated with the combination therapy showed a tendency to increased splenocyte response (Figure 6S), and an increase in antibody response (Figure 6T). The results show that the R-337 and anti-PD-1 combination therapy was highly effective and are consistent with the view that the distant protection was immune-based.

### 4.5. Retargeted oHSVs Eradicate High Grade Gliomas (HGG) in Preclinical Models

Glioblastomas (GBMs) are among the tumors with highest resistance to surgery, chemo- and radiotherapy, and highest mortality rate. Essentially, the natural history of these tumors has not changed in the last 50 years. GBMs have been the subject of intense interest as targets of OV-based therapy, and especially of oHSVs, in part because of the natural tropism of HSV for the nervous system. Human GBMs express TAAs, such as HER-2, EGFRvIII, IL-13R2α, EGFR and others.

We provided proof of principle that GBM can potentially be treated with retargeted oHSVs. In initial studies, Malatesta and his group developed a high-grade glioma (HGG) model, consisting of human GBM cells genetically modified to express HER2 and orthotopically implanted in the brains of immunodeficient mice [57]. When the 1st generation unarmed R-LM113 was administered i.t. as single dose, it more than doubled the survival time, and fully protected about 20% of the mice [57]. These findings confirmed and extended similar finding on EGFRvIII-expressing GBM cells [49]. Subsequently, the Malatesta group developed a genetically engineered HGG preclinical model in immunocompetent syngeneic BALB/c mice. The tumors cells were derived upon overexpression of platelet-derived growth factor B (PDGF-B). This model exhibits a gene expression profile typical of oligodendrocyte precursor cells and histopathological features typical of GBM, thus recapitulating GBM [66]. The cancer cells were made transgenic for HER2, and orthotopically implanted in the brains of BALB/c mice. A single dose of R-115 administered intracranially (i.c.) fully protected 30% of mice. At sacrifice, the protected mice did not harbor any remnant of tumor. Interestingly, as we observed with the LLC-1-HER2-bearing mice, all the R-115-treated mice which survived the primary tumor exhibited a long-term distant protection and developed immune response to the tumor. This consisted of a systemic IgG response, as well as of a local response, whereby tumors became infiltrated with CD4+ and CD8+ lymphocytes [60].

### 4.6. Efficacy of Systemically Administered Retargeted oHSVs

A major aim in the OV field has been the development of agents suitable for systemic treatment of metastatic cancer. Given the natural history of the viruses from which the different OVs are derived, some OVs are better suited than others for systemic administration. In addition to unspecific uptake by parenchymal organs—a barrier for all OVs, the OVs based on some human viruses, like HSV, human Adenoviruses, measles viruses need to contrast the prior immunity that exists in humans. The question arose whether retargeted oHSVs are sufficiently robust for systemic delivery. In early studies we found that a 1st generation retargeted oHSV, named R-LM249, administered by weekly i.p. injections, significantly reduced brain and ovarian metastases by HER2-positive human tumors in immunodeficient mice carrying multiorgan tumors [56]. R-LM249 could also be delivered by means of mesenchymal carrier cells and decreased metastatic lung burden [58]. Most recently, in a immunocompetent mouse model that takes advantage of the immunotherapeutic effect, a 1st generation oHSV payloaded with IL-12 and GM-CSF, and administered in combination with anti-PD1, was capable of strongly decreasing the burden of lung tumor nodules induced by intravenous administration of LLC-1-HER2 cells, a model of metastatic lung disease [21]. Remarkably, the tumor growth inhibition occurred also in HSV-preimmunized mice [21]. The finding provides evidence that appropriately armed retargeted oHSVs in combination with checkpoint blockade can be a suitable agent for systemic administration.

### 4.7. Patents

Herpes Simplex Virus (HSV) with modified tropism, uses and process of preparation thereof—WO2009144755 (divisional patent EP2700405)

Retargeted herpesvirus with a glycoprotein H fusion—WO201612849

Herpesvirus with Modified Glycoprotein B—WO2017211941

Herpesvirus with Modified Glycoprotein D—WO2017211944

Herpesvirus with Modified Glycoprotein H For Propagation In A Cell—WO2017211945

## 5. Conclusions

The main features to emerge from current review on retargeted oHSVs are as follows.

A major argument that may be raised against tropism retargeted oHSVs is that they are not anti-pan-tumor agents. This argument does not take into account that any selected TAA is expressed across a number of cancer types, and therefore any retargeted oHSV can potentially be employed against a variety of indications. Moreover, clinical practice indicates that, due to cancer heterogeneity, the therapeutic effects of potentially pan-tumor OVs is not uniformly exerted on any type of tumor, or of cancer patient. The current trend in anti-cancer therapy is to develop therapeutic agents tailored on the patient characteristic, since it is futile and even counterproductive to administer a therapy to a patient who will not benefit of it (see reference [67] in this issue). The retargeted oHSVs meet this need. It is interesting to note that the restriction to predefined targets is shared with CAR-Ts (chimeric antigen receptor T cells) to solid tumors, which are essentially addressed to the same targets as the retargeted oHSVs [68,69]. The retargeted oHSVs and CAR-Ts could potentially be combined, and the combination could overcome some of the limits encountered by CAR-Ts, e.g., the difficulty to populate the tumors and to overcome the TME immunosuppression, essentially hostile to lymphocyte proliferation and activation [70].

A second Con is that the retargeted oHSVs strictly depend on the presence of the targeted receptor for infection, also for infection of producer cells. This property greatly limits the repertoire of cells which can be employed for production. Inasmuch as it advisable to make use of non-cancerous cells for clinical grade virus production, we set-up a strategy for virus growth, based on a double retargeting. Finally, it has long been debated that a disadvantage of employing human viruses as OVs is the prior immunity, which can block virions, particularly if they are administered by systemic routes. This notion should be reassessed in light of the interesting discovery that prior immunity, or an unrelated immune response, can actually contribute to unleashing the TME immunosuppression [71]. Indeed, the high efficacy deployed by the retargeted oHSVs enabled a systemic delivery, even in mice carrying a prior immunity to HSV [21]. In the field of vaccines to infectious agents, an analogous potentiation of the innate response by immunologically unrelated priming was also reported [72]. Whether such effects apply to humans treated with HSV-based OVs remains to be determined.

On the Pro side, the IL-12-positive R-337 was highly effective in eradicating the primary tumor, modifying the TME, and eliciting an antigen-agnostic long-term anti-tumor vaccination. How do we explain the high abscopal efficacy and antigen-agnostic vaccination? It is well known that wt-HSVs cause immunogenic cell death and are inducers of innate response, which they blunt later in infection [35,36,37,38]. Typical features of the innate response to HSV are the secretion of IFN-α, -β, and -γ—through STING and other sensors—and of additional pro-inflammatory cytokines, infiltration by and activation of NK cells, of CD4+ and CD8+ lymphocytes, and of dendritic cells (DCs). The high levels of IFN-γ and the recruitment and activation of DCs, T and NK cells are hallmarks of the early phases of innate and adaptive responses to the tumor. Thus, the innate antiviral response may well serve as the switch that turns on the anti-tumor innate response; the latter then evolves into the adaptive response [73]. The LLC-1-HER2 tumor employed in current studies exemplifies a tumor insensitive to anti-PD-1. Treatment with R-337 efficiently unleashed the resistance, such that the efficacy of both R-337 and anti-PD-1 were dramatically increased in a combination regimen and resulted in high protection. Thus, the retargeted oHSVs appear to serve well the function of augmenting the tumor sensitivity to CPIs.

In mice there was no detectable off-tumor and off-target infections, even though the potential of spread to tissues with low level expression of the targeted receptor (HER-2, in our case) remains to be verified in humans for any given retargeted oHSV.

In conclusion, major difficulties facing the clinical translation of retargeted oHSVs to the clinic have been tackled and solutions have been found. The stage is ready for further developments and for verification at the bedside of how well this class of therapeutic agents will hold promise.

## Figures and Tables

**Table 1:** 1st, 2nd and 3rd generation retargeted oHSV, genotypic modifications for retargeting and detargeting purposes.

Generation of Recombinant	Name of Recombinant	scFv for Retargeting to Tumor Cells Inserted	GCN4 Peptide for Retargeting to Producer Cells Inserted in	Detargeting Strategy, Deletions @ gD	Ref
1st	R-LM113 R-115 R-123	HER2 @ gD	Absent	Δ aa 6–38	[21,27,42]
	R-LM249	HER2 @ gD	Absent	Δ aa 61–218	[41]
	R-611	EGFR @ gD	Absent	Δ aa 6–38	[42]
	R-613	EGFRVIII @ gD	Absent	Δ aa 6–38	[42]
	R-593	PSMA @ gD	Absent	Δ aa 6–38	[42]
2nd	R-803 R-809	HER2 @gH	Absent	No deletion, or Δ aa 6–38	[43]
	R-903 R-909	HER2 @ gB	Absent	No deletion, or Δ aa 6–38	[44]
3rd	R-313, R-315 R-317 R-319	HER2 @ gD	@ gB	Δ aa 6–38	[46]
	R-213	HER2 @ gD	@ gH	Δ aa 6–38	[45]
	R-87 R-89 R-97 R-99 R-99-2	HER2 @ gD	@ gD	Deletions, various	[47]
	R-321, R-335 R-337	HER2 @ gD	@ gB	Δ aa30 and aa38	[46] this paper

**Table 2:** 1st and 3rd generation retargeted oHSVs. Summary of preclinical studies.

	Name of Recombinant	Payload	Preclinical Assays and Main Results							Ref
			Tumor	Mice	Efficacy against Primary Tumor	Efficacy against Distant Challenge Tumor	Modifications to TME	Combo with CPI	Route of Administration	
1st generation	R-LM249	None	Human Ovary SK-OV-3	Immune- deficient	Very high protection. Protection from disseminated metastases.	NT	NT	NT	I.T. I.P. Carrier cells	[41,56,58]
	R-LM113	None	Mouse lung (LLC-1-HER2)	Immune-competent, HER2-tolerant	Complete response against primary tumor in 30% mice in early treatment.	High protection	Low modifications	NT	I.T.	[55]
	R-LM113	None	Human high grade glioma AND Murine high grade glioma	Immune-deficient AND Immune-competent	Doubling in survival time AND Doubling in survival time	NT	NT	NT	I.C.	[57,59]
	R-115	mIL-12	LLC-1-HER2	Immune-competent, HER2-tolerant	Complete response against primary tumor in 70% mice in early treatment.	High protection	Infiltration by immune cells. Increased cytokines		I.T.	[55]
	R-115	mIL-12	Murine high grade glioma (HGG-HER2)	Immune-competent	Complete eradication in 30% mice in late treatment	High protection	Increased infiltration by CD4 CD8		I.C.	[60]
	R-123	mIL-12 + mGM-CSF	LLC-1-HER2	Immune-competent, HER2-tolerant	Complete response against primary tumor in 40% mice in late treatment.	NT		Full protection	I.T. I.V.	[21]
3rd generation	R-335	mIL-12	LLC-1-HER2	Immune-competent, HER2-tolerant	Complete response in 30% mice in late treatment	High protection	NT	Low potentiation	I.T.	This paper
	R-337	mIL-12 fusion protein	LLC-1-HER2	Immune-competent, HER2-tolerant	Complete response in 80% mice in late treatment	High protection	Increased infiltration by immune cells; increased expression of cytokines	Potentiation	I.T.	This paper

NT, not tested.

## Data Availability

Data is contained within current article or previous publications.
